# MXene-Decorated Nylon Mesh Filters for Improvement of Indoor Air Quality by PM_2.5_ Filtration

**DOI:** 10.1021/acsomega.3c00452

**Published:** 2023-06-21

**Authors:** Melek Hazal Baskoy, Oyku Cetin, Serkan Koylan, Yaqoob Khan, Gurdal Tuncel, Tuba Hande Erguder, Husnu Emrah Unalan

**Affiliations:** † Department of Environmental Engineering, Middle East Technical University (METU), 06800 Ankara, Turkey; ‡ Department of Metallurgical and Materials Engineering, Middle East Technical University (METU), 06800 Ankara, Turkey; § Quantum Solid State Physics (QSP), KU Leuven, Celestijnenlaan 220D, Leuven 3001, Belgium

## Abstract

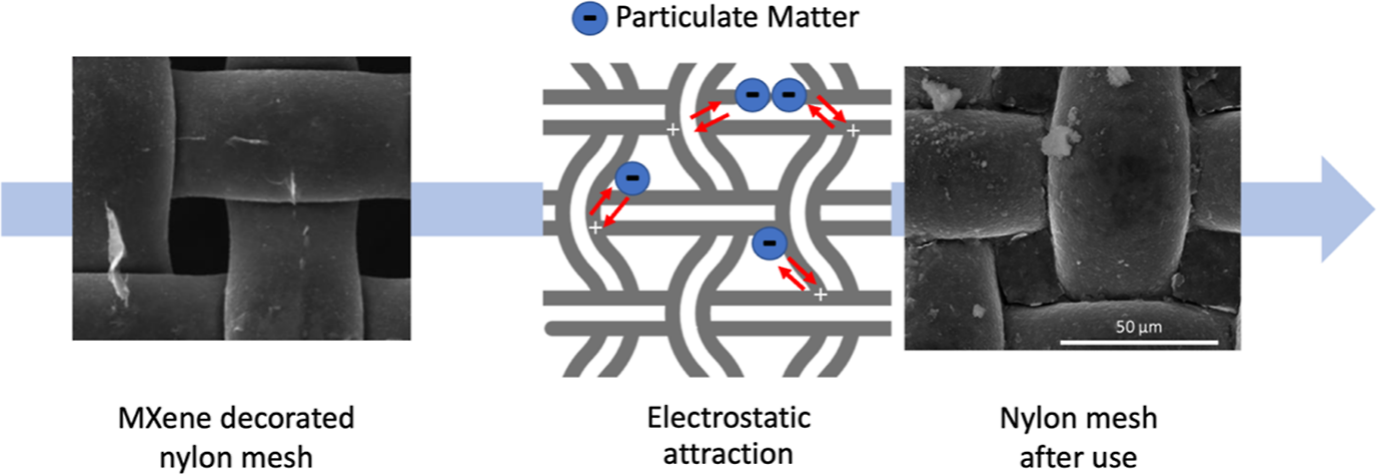

Air pollution is a problem that is increasing day by day and poses a threat on a global scale. Particulate matter (PM) is one of the air pollutants that is the biggest concern regarding air quality. In order to control PM pollution, highly effective air filters are required. This is especially necessary for PM with a diameter of less than 2.5 micrometers (PM_2.5_), which poses a health risk to humans. In this study, we demonstrate for the first time the use of a two-dimensional titanium carbide (Ti_3_C_2_) MXene nanosheets-decorated nylon mesh (MDNM) as a low cost and highly efficient PM_2.5_ filter. This study develops a proof-of-concept method to capture PM_2.5_. Thanks to their high specific surface area and active surface-terminating groups, conductive MXene nanosheets have made nylon mesh filters promising candidates for air filtration. The developed filters used electrostatic force to capture PM_2.5_ and showed high removal efficiency (90.05%) when an ionizer was used and under an applied voltage of 10 V, while a commercial high-efficiency particulate air (HEPA) filter had a removal efficiency of 91.03% measured under identical conditions. The proposed filters, which stand out with their low energy consumption, low pressure drop (∼14 Pa), and cost-effectiveness, have the potential to be a strong competitor to conventional PM filter systems used in many fields.

## 1 Introduction

The fast growth of megacities, globalization of industrial production, proliferation of pesticides and harmful chemicals, and increased use of motor vehicles raise the pollutant concentration in the air, known as air pollution. As air pollution expands, the possibility of inhalation of pollutant particles by living species also increases, making air pollution a significant threat to human health.^1−3^ Since 1990, the death rate from air pollution has been increasing globally, with the sharpest increases observed in the world’s fastest growing economies.^4^ Particulate matter (PM), which is one of the criteria pollutants affecting air quality, has various chemical and physical properties due to its particle size, regions, and the emission source. Airborne pollutants with an aerodynamic diameter of less than 2.5 μm are categorized as PM 2.5 (PM_2.5_), which is composed of small solid particles and liquid droplets made up of inorganic and organic chemical constituents. The harmful effects of PM_2.5_ on human health have been proven. PM_2.5_ is known to penetrate the human body through the respiratory system and cause various diseases, including different types of cancer.^5−11^ By 2060, the World Health Organization (WHO) estimates that 6–9 million people will die due to air pollution.^12−15^ Considering the harmful effects of PM_2.5_ on human health, the importance of air filtration is increasing day by day.

Indoor air quality has become even more important, especially during the COVID-19 pandemic, as most people spend 80–90% of their time indoors.^16,17^ Indoor areas allow potential pollutants to build up more than outdoor areas, and the exposure time to indoor air pollution is longer. United States Environmental Protection Agency (USEPA) has stated that indoor air pollutant levels are usually 2 to 5 times higher than outdoor pollutant levels.^18,19^ Air filtration is considered a critical element of environmental control measures to improve air quality.^20^ Various methods have been developed for air filtration to reduce the PM_2.5_ concentration in the air and its potential hazards to human health. One of the most commonly used methods is the size-exclusion-based air filtration. In this type of filtration, solid particles are separated from air based on their sizes. Thereby, the desired particles are removed using a filter with appropriate pore size. Porous membrane filters work based on the exclusion principle. In porous membranes, pore sizes should be designed in such a way that they successfully prevent transition of unwanted particles through the membrane filter.^21^ However, the as-collected particles, which plug up the pores at the entrance of the filter, significantly increase the air flow resistance and, as a result, the pressure drop between the inlet and the outlet of the filters increases.^22^ The other PM_2.5_ filtration technique is based on the adsorption of PM through surface–particle interactions.^23^ Microfiber-based and nanofiber-based filters are typically used in the adhesion-related filtering method.^24^ The basic working principle depends on the surface functionalization of the filter material to enhance surface–particle interactions by a proper intermolecular force optimization. The adsorption method also imparts antibacterial properties due to the use of fiber-based filters, which has become even more important during the COVID-19 pandemic.^25,26^ Commercially available filters with the surface–particle interaction mechanism are widely used in public places such as hospitals, shopping malls, and transportation vehicles, where long-term utilization of the filter is necessary without replacement. However, the adhesion-related filtering method suffers from high airflow resistance, which results in a high pressure drop. Powerful pumps, which can be expensive, are required to generate sufficient air flow to overcome high pressure drop across the filter. Thus, efficiency decreases, while energy consumption increases. Because micron-sized fibers are used as the filter material, it is difficult to maintain high-efficiency PM_2.5_ filtration cost effectively over its long service life.^27,28^

Increasing the surface area of the filter may enhance the effective use of the surface functional groups (−F, −H, and −OH) on the filter structure.^29^ Enhancement in filtration efficiency with the introduction of surface functional groups is a simpler and cost-effective method to capture PM_2.5_ than trying to find a solution for the pressure drop problem of size-exclusion-based filters.^30−32^ Instead of filtering the air by the size exclusion method, filtering PM_2.5_ from the air with filter–PM interactions using a proper backbone helps to overcome the pressure drop problem. By using 2-dimensional (2D) nanomaterials in filters making use of the adhesion-related filtration method, the high filter surface area can be achieved to overcome the problems related to the pressure drop.^33−35^

The successful use of Ti_3_C_2_ MXene in filtration is demonstrated with examples ranging from desalination/water treatment to gas separation.^36,37^ Ti_3_C_2_ MXene is a promising 2D material with a high concentration of surface functional groups (−F, −H, and −OH). This makes it a candidate material for adhesion-related indoor air filtration applications.^38−40^ During the chemical delamination step, the surface-terminating groups bond to the surface of MXene nanosheets.^41,42^ These surface-terminating groups form strong interactions not only with PM_2.5_ but also with bacteria, improving the indoor air filtration performance of the filter material.^43^ For instance, Gao et al. (2019)^31^ showed that the MXene nanosheets inhibit the propagation of bacteria (e.g. *Escherichia coli* and *Staphylococcus aureus*) that are part of respirable microorganisms in airborne PM. Besides the fact that MXene is antibacterial, studies also reported that MXene synthesis is becoming an environmentally friendly process with alternative precursors and production methods^44,45^ followed by biodegradable products,^46^ which coincides with the trend of producing environmentally friendly air filters in recent years.^47^

Filtration materials and the filtration mechanism have a significant impact on the filtration process. In recent years, many types of filtration materials including polymers, metal–organic frameworks, carbon-based materials (i.e., carbon nanotubes), silk, chitosan, oxides, and nanowire networks, have been proposed for PM capture.^5,24,48−52^ Conventional air filters use mechanisms such as gravity, inertia, interception, and diffusion to capture the particles.^21^ In order to produce an efficient filter, multiple filtration mechanisms should be utilized by taking the particle size and density, fiber thickness, and air velocity into consideration. Conventional air filters can passively provide high PM removal efficiency with the help of small pores, but as more particles are trapped, air permeability, another important parameter in filter evaluation, decreases. Therefore, the electrostatic air filtration method is more prominent in the collection of particles of all sizes. The basic working principle in this method is the electrostatic attraction of the electrically charged particle and the electrically oppositely charged filter material.

The air purification performance of nanomaterials has improved over the years. Many different filter materials such as polyester, nylon, cotton etc., are developed with nanomaterials such as graphene and silver nanoparticles and nanowires to capture PM. Outstanding studies in the literature on the use nanomaterials in air purification are summarized and presented in Table 1. For the first time, Jeong et al. (2017)^33^ developed a silver nanowire (Ag NW)–nylon system that can be biased to collect PM by electrostatic forces. This type of filtration system has several advantages such as high antibacterial performance and reusability. Then, Huang et al. (2019)^53^ designed a flexible, transparent, and stable Ag-nylon mesh that can be biased as well for PM_2.5_ removal. The cost of fabrication was reported as $15.03, and the fabrication took only 20 min. Zhang and Hsieh (2020)^54^ designed a simple, low-cost, versatile, and scalable electrostatic air filter with the help of Ag NWs and titanium dioxide (TiO_2_) nanoparticles for PM removal and formaldehyde decomposition. Lian et al. (2020)^55^ proposed a multifunctional electronic textile (e-textile) based on cotton substrates decorated with Ag NWs for different purposes including PM_2.5_ filtration. In a very recent study, Narakaew et al. (2022)^56^ utilized Ag NW bamboo-charcoal composition networks on nylon sheets for PM removal by electrostatic and intermolecular forces. Liu et al. (2015)^57^ demonstrated that electrospun polyacrylonitrile (PAN) nanofibers can be highly effective PM filters. Zhang et al. (2016)^24^ developed highly efficient polyimide-nanofiber air filters for PM_2.5_ removal. Han et al. (2021)^52^ utilized copper nanowire (Cu NW)-decorated air filters for the effective capture of PM by mechanical and electrostatic filtration mechanisms. Jung et al. (2018)^5^ demonstrated highly efficient PM filters that were decorated with reduced graphene oxide. Although there has been increased interest in using nanomaterials for air filtration, there is only one study on the utilization of 2D MXene for air filtration, where MXene nanosheets were incorporated into PAN fibers during the electrospinning method to obtain the filters. A PM_2.5_ removal efficiency of 99.7% with a pressure drop of 42 Pa was reported. Moreover, 2D MXene nanosheets-incorporated PAN filters were also demonstrated to effectively inhibit bacterial growth.^31^

**Table 1:** Recent Studies on Air Filtration through the Use of Nanomaterials

nanomaterial	backbone	fabrication method	results	refs
Ag NWs	nylon mesh	vacuum filtration	PM_2.5_ removal efficiency: >99.99% pressure drop: 3.51 Pa	(33)
Ag NWs	nylon mesh	dip coating	PM_2.5_ removal efficiency: 99.65% pressure drop: 14.43 Pa	(53)
TiO_2_-decorated Ag NWs	nonwoven polyester fabric	layer-by-layer assembly, electrophoretic deposition, and dip coating	PM_2.5_ removal efficiency: 99.5% pressure drop: 11 Pa	(54)
Ag NWs	cotton	dip coating	PM_2.5_ removal efficiency: >98%	(55)
Ag NWs/bamboo charcoal composite	nylon mesh	dip coating	PM_2.5_ removal efficiency: >99.9%	(56)
PAN nanofibers	fiber glass wire mesh	electrospinning	PM_2.5_ removal efficiency: >95% pressure drop: 133 Pa	(57)
polyimide nanofibers	copper mesh	electrospinning	PM_2.5_ removal efficiency: >99.5% pressure drop: 73 Pa	(24)
Cu nanowires	nylon mesh	vacuum filtration	PM_2.5_ removal efficiency: 99.3% pressure drop: 25–150 Pa	(52)
reduced graphene oxide	copper mesh	ion-mediated assembly	PM_2.5_ removal efficiency: >99.9% pressure drop: 5 Pa	(5)
MXene	PAN	electrospinning	PM_2.5_ removal efficiency: ∼99.7% pressure drop: ∼42 Pa	(31)

In this work, we developed unique Ti_3_C_2_ MXene-decorated nylon mesh (MDNM) filters for indoor PM_2.5_ removal from the air. Most of the studies^31,33,53,54,56^ concerning air filtration materials utilize an additional PM source to create a pollutant environment. In this study, without using an extra PM source, the indoor air was drawn into a filtration setup by a fan. Since more PM can be captured with a large-sized filter, MDNM filters were designed with dimensions of 14 cm × 14 cm. The filters had a simple geometry and were fabricated through a cheap, simple, and rapid dip-coating method. The nylon mesh acted as the backbone structure of the filter where upon decoration, 2D MXene flakes showed promising performance in PM removal, thanks to their high electrical conductivity and large surface area. Nylon mesh has wide openings, is readily available, inexpensive, and lightweight. For all these reasons, it was chosen as a filter material in this study. Although nylon mesh is a material that is frequently coated with nanomaterials, its use in air filtration upon MXene decoration has not been investigated yet. In this study, a MDNM filter was used for high-efficiency capture of PM_2.5_. To create an electrostatic attraction force, the PM was negatively charged by the ionizer, while the MDNM filter was positively charged by the applied voltage. Low-pressure drop-in operation combined with low-cost and readily available starting materials and a highly scalable production route compared to previously reported air filtration approaches make the proposed filters highly suitable for PM_2.5_ filtration.

## 2 Experimental Procedure

### 2.1 Fabrication of MDNM Filters

#### 2.1.1 Synthesis of Nanomaterials

##### 2.1.1.1 Preparation of Ti_3_AlC_2_ MAX Phase

**Figure 1:**
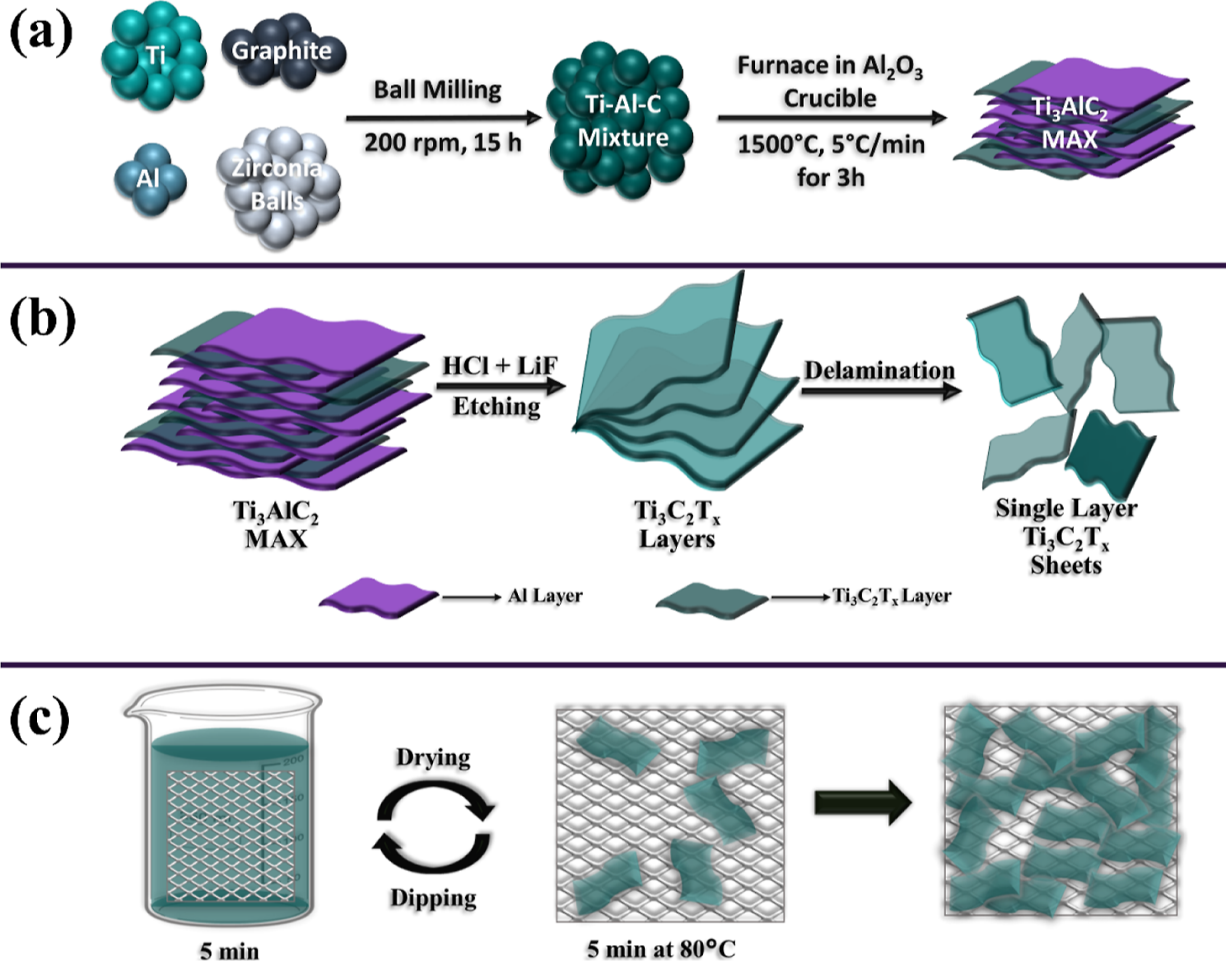
Schematics of (a) fabrication process of MAX phase, (b) fabrication process of MXene nanosheets, and (c) preparation route of MDNM filters through dip-coating.

The starting raw materials were commercially available Ti (Micron powder, Purity: 99.9%, Nanografi, Turkey), Al (Micron powder, Purity: 99.99 percent, Nanografi, Turkey), and graphite (Fisher Scientific, USA) powders. The stoichiometric ratio of Ti, Al, and C was 3:1:2. The powder mixture was ball-milled for 15 h in a Teflon container using 5 mm zirconia milling balls (Retsch PM100) with a ball to powder ratio of 4:1. The milled mixture was loaded in a Al_2_O_3_ crucible and placed in a tube furnace with continuous argon flow. The furnace was set to 1500 °C with a heating rate of 5 °C/min, and the mixture was kept at 1500 °C for 3 h. Following the cooling of the furnace, the MAX phase was collected and crushed using a bench drill (Bosch, PBD 40) to obtain Ti_3_AlC_2_ particles in a powder form. The preparation route of the MAX phase is schematically illustrated in Figure 1a. SEM image of the Ti_3_AlC_2_ MAX phase produced and used in this work is provided in Figure S1.

##### 2.1.1.2 Preparation of Ti_3_C_2_ MXene Nanosheets

The MXene nanosheets were obtained through the liquid exfoliation method which is schematically shown in Figure 1b. In a typical fabrication, 3.1 M lithium fluoride (LiF) (Sigma-Aldrich) solution was prepared in 40 ml of 9 M hydrochloric acid (HCl) (Sigma-Aldrich) solution in a polytetrafluoroethylene (PTFE) beaker. Then, 2 g of Ti_3_AlC_2_ MAX powder was added slowly to the prepared solution, and the mixture was stirred at 350 rpm at 35 °C for 24 h. The resulting products were washed several times with deionized (DI) water (18.3 MΩ) and centrifuged at 4000 rpm until the pH of the dispersion reached 6. The delaminated MXene nanosheets were separated from the unreacted MAX phase by centrifugation at 4500 rpm, and the fabricated MXene nanosheets were collected from the supernatant. MXene nanosheets from the supernatant were concentrated using centrifugation at 10,000 rpm for 10 min, and then the concentration of MXene nanosheets-DI water dispersion was adjusted to 5 mg/mL for further deposition process and characterizations.

#### 2.1.2 Coating of MDNM Filters

14 cm × 14 cm nylon meshes with a pore size of 20 μm were precleaned via ultrasonication using acetone, ethanol, and DI water for 10 min at each step. Next, nylon meshes were dipped into concentrated DI water–MXene dispersion for 5 min and then dried in a furnace at 80 °C. The dipping and drying cycles were repeated until homogeneous MDNM filters were obtained. A multimeter (TENMA 72-7730) was used to check the resistance of filters. The preparation procedure of MDNM filters is schematically illustrated in Figure 1c.

### 2.2 Performance Assessment of MDNM Filters

**Figure 2:**
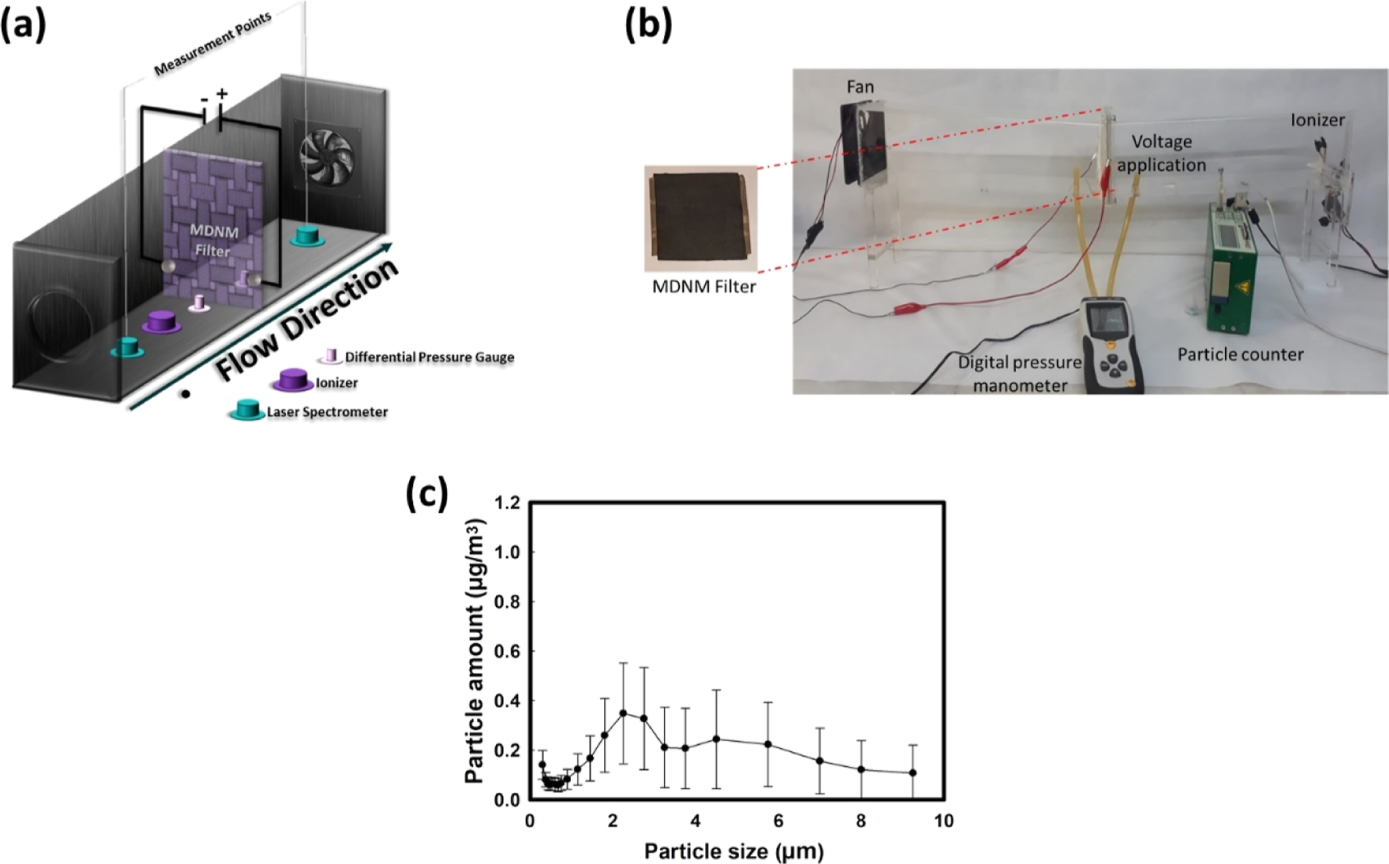
(a) Schematic of the air filtration setup, (b) photograph(s) of the air filtration setup and MDNM filter, and (c) size distribution of the particles in the indoor air according to mass concentration.

Several experiments were performed to determine the filtration performance of the MDNM filters. Filtration tests had to be carried out in a calm and steady room using an airtight setup. Therefore, a filtration setup was designed and fabricated in-house. All filtration tests were performed under the same temperature (24.0 ± 1.0 °C) and relative humidity (35–40% ± 2%) conditions as measured by a laser aerosol spectrometry (LAS) device. A schematic illustration and photograph(s) of the filtration setup with MDNM filter are given in Figures 2a,b, respectively. This setup was designed to test both conductive nanomaterial-decorated filters and high-efficiency particulate air (HEPA) filter. All measurements were also made using a reference HEPA filter (AFT Filter, Micro Glassfiber). According to EN 1822 standards, this HEPA filter is in the filter class of H13. This HEPA filter has a removal efficiency of 99.95% as stated in the technical specifications’ datasheet (Figure S2).

As shown in Figures 2a,b, the filtration setup contained two divided plexiglass chambers, consisting of a fan, particle counter, power supply, pressure gauge, and an ionizer. The MDNM filter was located in the middle of the two chambers with a plexiglass frame. In this way, the filters could be easily changed without changing the air tightness of the setup. A fan (Taida-Tidar) was installed to provide sufficient air flow to the setup. Most of the studies performed with different air filtration materials utilize an additional PM source, such as a burning incense, to simulate a polluted environment.^5,33,56^ In this study, the indoor air was drawn by a fan without using an extra PM source. At the inlet part of the chamber, an ionizer (TRUMPXP, TFB-YD1249) was located to negatively charge the PM. The particles in both chambers were counted via the LAS device called Grimm Environmental Dust Monitor (EDM) 107. An anemometer (ALMEMO, 2290-4) was used for measuring the airflow velocity. A two-channel power supply (TT TECHNIC MCH305D-II) was used to both apply voltage and change the fan speed. A digital differential pressure manometer (CEM, DT-8890) was used to detect the pressure drop during filtration.

Since there was only one particle counter for this study, the inlet PM measurement was performed when the air at the inlet measurement point of the setup became stable, and then PM measurements were performed at the outlet measurement point of the setup. The laboratory used for the filtration tests was specifically arranged to have no significant change in the indoor air. Indoor air was not disturbed by any other means, and all filtration tests were performed under similar conditions using actual indoor air. Yet, particle size distribution can affect the actual efficiency, especially when the particle size distributions are different at different concentration levels. The size distributions of the particles in the indoor air during the filtration tests performed in this study are given in Figure 2c.

These filtration tests were performed to investigate the effect of voltage application, the effect of air velocity, the effect of repeatability, and the effect of 2 h filtration performance on both the removal efficiency and pressure drop. SEM images of the MDNM filter after filtration test are provided in Figure S3.

The removal efficiencies of particles from 0.3 to 2.5 μm were calculated in this study since the most penetrating particle size (MPPS) is specified as 0.3 μm in the efficiency tests of HEPA filters according to the EN1822 standard.^58^ Input and output concentration measurements were used while calculating removal efficiencies according to the following equation
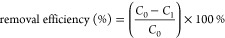
1where *C*_0_ (μg/m^3^) and *C*_1_ (μg/m^3^) denote the mass of PM in all sizes at the inlet and outlet measurement points of filtration setup, respectively.

There are two ways to measure the amount of PM in the atmosphere, namely mass concentrations and number concentrations. The PM counter, Grimm EDM107, used in this study gives the data in a count distribution mode (number concentration), which indicates the number of particle concentrations per liter for all particle measuring channels. However, mass concentration is used to indicate PM levels, since current standards are based on mass concentrations. Converting the particle amount to the mass concentration relies on two assumptions used in most particle sizing techniques. Accordingly, all particles are assumed to have a uniform density and spherical shape. In this study, number concentration was converted to mass concentration (Figure S4), and all data presented here are given as mass concentration.

### 2.3 Characterization of MDNM Filters

Different methods were used during the fabrication and the performance assessment of the filters decorated with MXene.

X-ray diffraction analysis was conducted using a Rigaku D/Max-2000 diffractometer with Cu Kα radiation at an operating voltage of 40 kV within a 2θ range of 5–70° at a scanning rate of 1°/min. The morphological analysis of the fabricated MXene nanosheets was made using transmission electron microscopy (TEM) performed at 200 kV in the high-resolution mode (HR-TEM) (JEOL JEM-2100F UHR/HRP 200 kV). The TEM sample was prepared by drop-casting the MXene-DI water dispersion onto holey carbon-coated 400 mesh copper grids followed by air-drying. The elemental maps of the MDNM filters were obtained through a field emission scanning electron microscope (Nova NanoSEM 430) at an operating voltage of 10 kV. X-ray photoelectron spectroscopy (XPS) was performed using a SPECS PHOIBOS instrument equipped with a hemispherical energy analyzer and an Al Kα X-ray source (14 kV, 350 W). In order to understand the adherence of the deposited MXene on the nylon mesh, bending and Scotch tape tests were performed on MDNM filters. The bending test was conducted using a custom-made bending machine, and the Scotch tape test was performed using 3M Scotch tape (Figure S5). A Keithley 2400 SourceMeter was used to monitor the change in the resistance of filters during the test.

## 3 Results and Discussion

### 3.1 Results of MDNM Filter Fabrication

**Figure 3:**
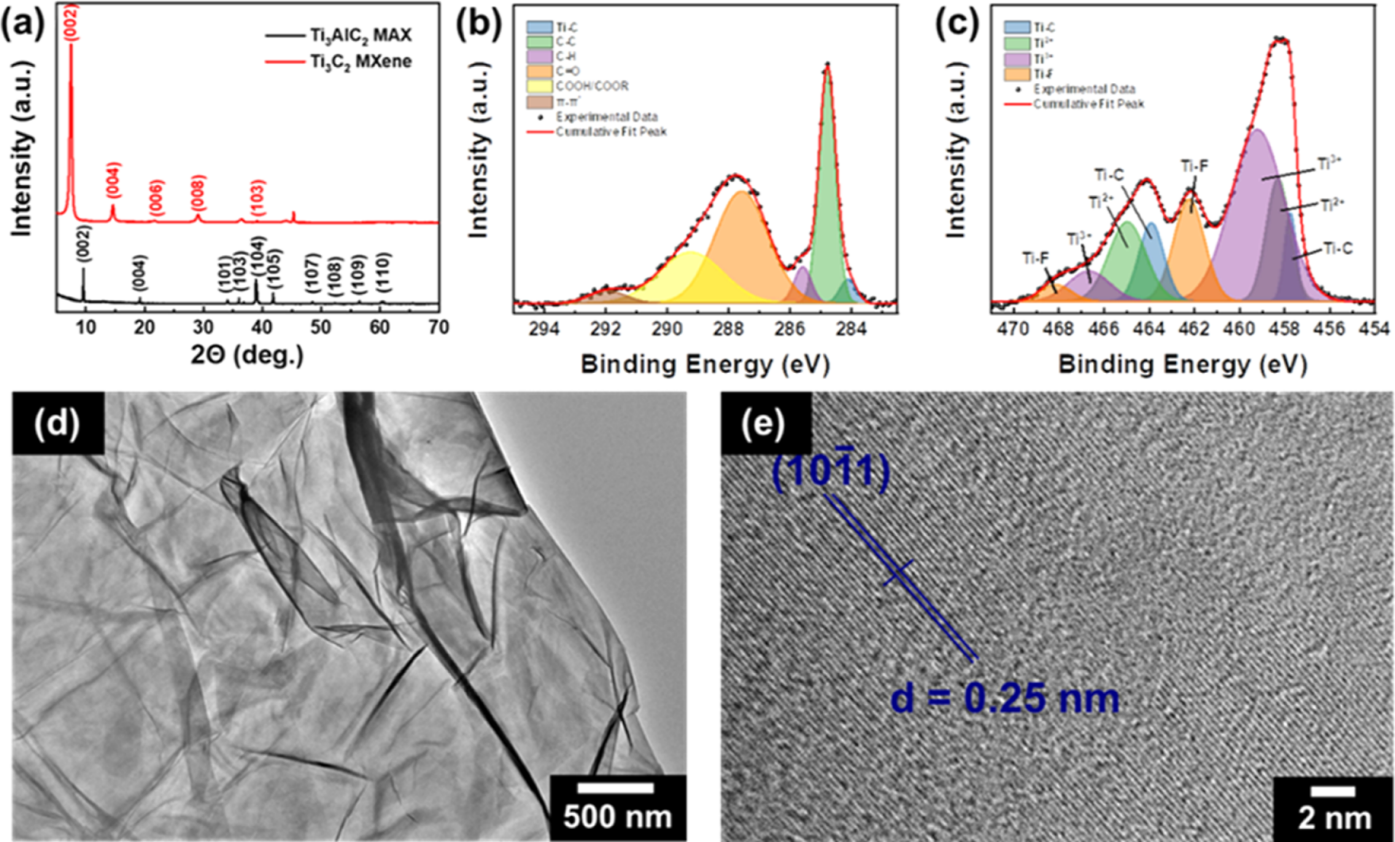
(a) XRD patterns of MAX phase and MXene nanosheets. High resolution XPS spectra of (b) C 1s and (c) Ti 2p regions, (d) BF-TEM image, and (e) HR-TEM image of MXene nanosheets with calculated *d*-spacing.

XRD patterns of the Ti_3_AlC_2_ MAX phase and Ti_3_C_2_ MXene nanosheets are provided in Figure 3a. The peaks of the MAX phase were sharp and belong to single-phase Ti_3_AlC_2_, which suggested a well-crystallized and pure material. The (002) plane of Ti_3_AlC_2_ phase occurred at 9.6°. On the other hand, the peak located at 7.5° corresponded to the (002) plane of the Ti_3_C_2_ phase. The small shift in the diffraction angle for (200) plane indicated successful removal of Al from the MAX phase and exfoliation of MXene nanosheets. In addition, the diffraction peaks of the as-fabricated MXene nanosheets were broader than those of the MAX phase due to the size effect. It can also be concluded that high-purity MXene nanosheets were produced, as no peaks were formed for the MAX phase in the XRD pattern of MXene nanosheets.

The MXene nanosheets used for the decoration of the nylon meshes were analyzed using high-resolution XPS. In the C 1s region shown in Figure 3b, six peaks were observed at 284.1, 284.8, 285.6, 287.6, 289.2, and 291.2 eV representing the Ti–C, C–C, C–H, C=O, COOH/COOR, and π–π* bonds, respectively. The Ti–C signal arises due to the formation of subjected bonds in Ti_3_C_2_ MXene after the etching of Al from the parent Ti_3_AlC_2_ MAX phase.^59^ Signals from the COOH/COOR are from oxygen binding to the carbon bonds, which are not fully formed while the C–O signal represents the MXene oxidation where TiO_2_ formation occurred.^60,61^ The Ti 2p region of the spectra provided in Figure 3c showed peaks corresponding to the Ti–C bond at 457.7 and 463.9 eV, Ti^2+^ at 458.3 and 465.0 eV, Ti^3+^ at 459.2 and 466.7 eV, and Ti–F bond at 462.2 and 468.1 eV. Ti^2+^ and Ti^3+^ signals are originated from mixed oxides and carboxides. The Ti–F bond signal is due to the surface functional group of Ti_3_C_2_ MXene, which originated from the chemical etching of the Ti_3_AlC_2_ MAX phase with HCl and LiF.^61,62^ The binding energies obtained from the XPS analysis of Ti_3_C_2_ MXene with the mentioned functional groups were found to be consistent with the recent MXene studies in the literature.^59,61,63^

A bright-field (BF) TEM image of MXene nanosheets is provided in Figure 3d to visualize the morphology of the as-fabricated Ti_3_C_2_ MXene. The transparent Ti_3_C_2_ flakes were crumpled due to the very low thickness and large flake sizes of the single-layer MXene. It was possible to observe folded and imbricated MXene flakes in the BF-TEM image due to the low contrast between MXene nanosheets. The HR-TEM image (Figure 3e) revealed the lattice fringes of the Ti_3_C_2_ crystal. The lattice spacing between the () plane of hexagonal Ti_3_C_2_ crystal was calculated as 0.25 nm, which is well-consistent with the literature.^64^

**Figure 4:**
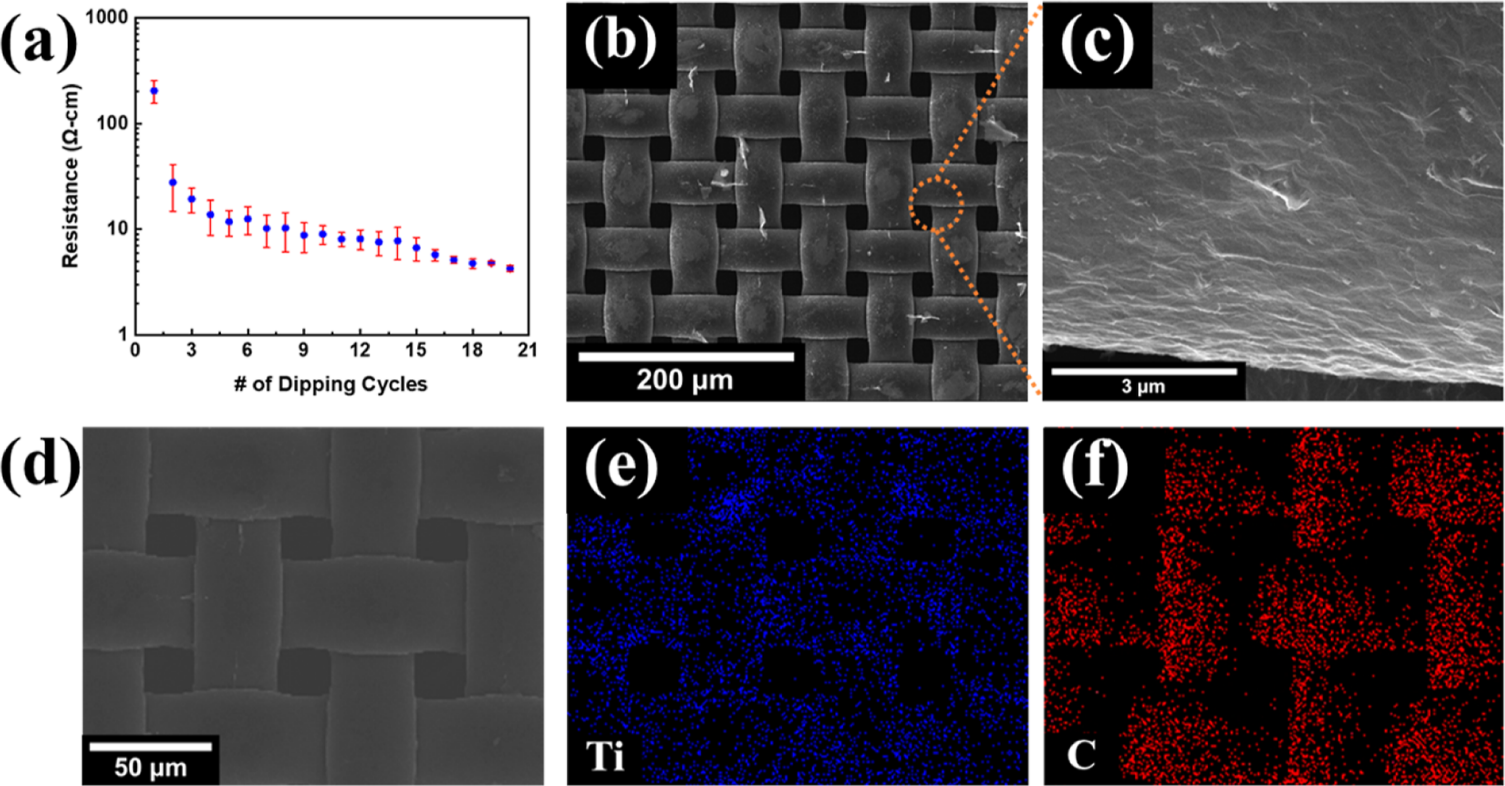
(a) Change in the resistance of MDNM filter with number of dipping cycles. (b) SEM image of a large area MDNM filter. (c) High-magnification SEM image of MDNM filter and (d) SEM image of MDNM filter for elemental maps and corresponding maps for (e) Ti and (f) C.

Figure 4a shows the change in the resistance of the fabricated MDNM filter with the number of dipping and drying cycles. Due to the high concentration of MXene-DI water suspension and large MXene flake size, it was possible to achieve a conductive network within few dip–dry cycles. Upon 20 dip–dry cycles, the resistance of MDNM filter was found to decrease below 10 Ω cm, with a clear reduction in standard deviation. Such rapid formation of the conductive network in a reproducible manner can certainly facilitate the mass production of MDNM filters. Deposition of MXene onto nylon mesh was monitored through periodic SEM analysis. The SEM image of a bare nylon mesh is given in Figure S6. Low- and high-magnification SEM images of MDNM filters with a resistance of 3.86 Ω cm are provided in Figures 4b,c, respectively. All fibers forming the nylon mesh were conformably decorated with MXene nanosheets. As no electron charging occurred during the SEM analysis, there was no non-conductive part in the large-area coating, indicating that MXene was uniformly coated on the nylon mesh. This point was also confirmed by the areal resistance measurements. When a single fiber coated with MXene nanosheets was examined in Figure 4c, the surface of the fiber was found to be covered with MXene nanosheets, and they formed a conductive network by overlapping with each other. In addition, no structural deformation was observed for the nylon mesh, and there was still a 20 μm opening between the fibers. This was necessary to maintain a low pressure drop during PM_2.5_ filtering using the fabricated MDNM filters. Figure 4d shows an SEM image of MDNM and corresponding EDS maps for titanium (Ti) (Figure 4e) and carbon (C) (Figure 4f) to ensure the homogeneous distribution of MXene nanosheets. The EDS elemental maps showed that the Ti and C signals collected from the surface of the fibers have a similar shape to that of nylon mesh. The change in the resistance values during the bending test and Scotch tape test are provided in Figures S5a,b, respectively. After 1000 bending cycles down to a bending angle of 80°, only a 10% increase in the resistance of MDNM filters was observed. The Scotch Tape test was repeated 10 times, during which there was no significant change in the resistance of the MDNM filter. The results revealed that, even without an overlayer, the adhesion of the MXene nanosheets to the nylon mesh appeared strong enough to prevent any significant loss in electrical conductivity as a result of any external interference.

### 3.2 Results of Performance Assessment Tests

There are some critical parameters that must be met to evaluate the performance of filtration materials, especially in their practical applications. Among these parameters, removal efficiency is regarded as the most important one. The following sections discuss the effects of predetermined factors, namely, voltage application, air velocity, repeatability, and 2 h filtration on performance of filtration material.

#### 3.2.1 Effect of Voltage Application on the Filtration Performance of MDNM Filters

**Figure 5:**
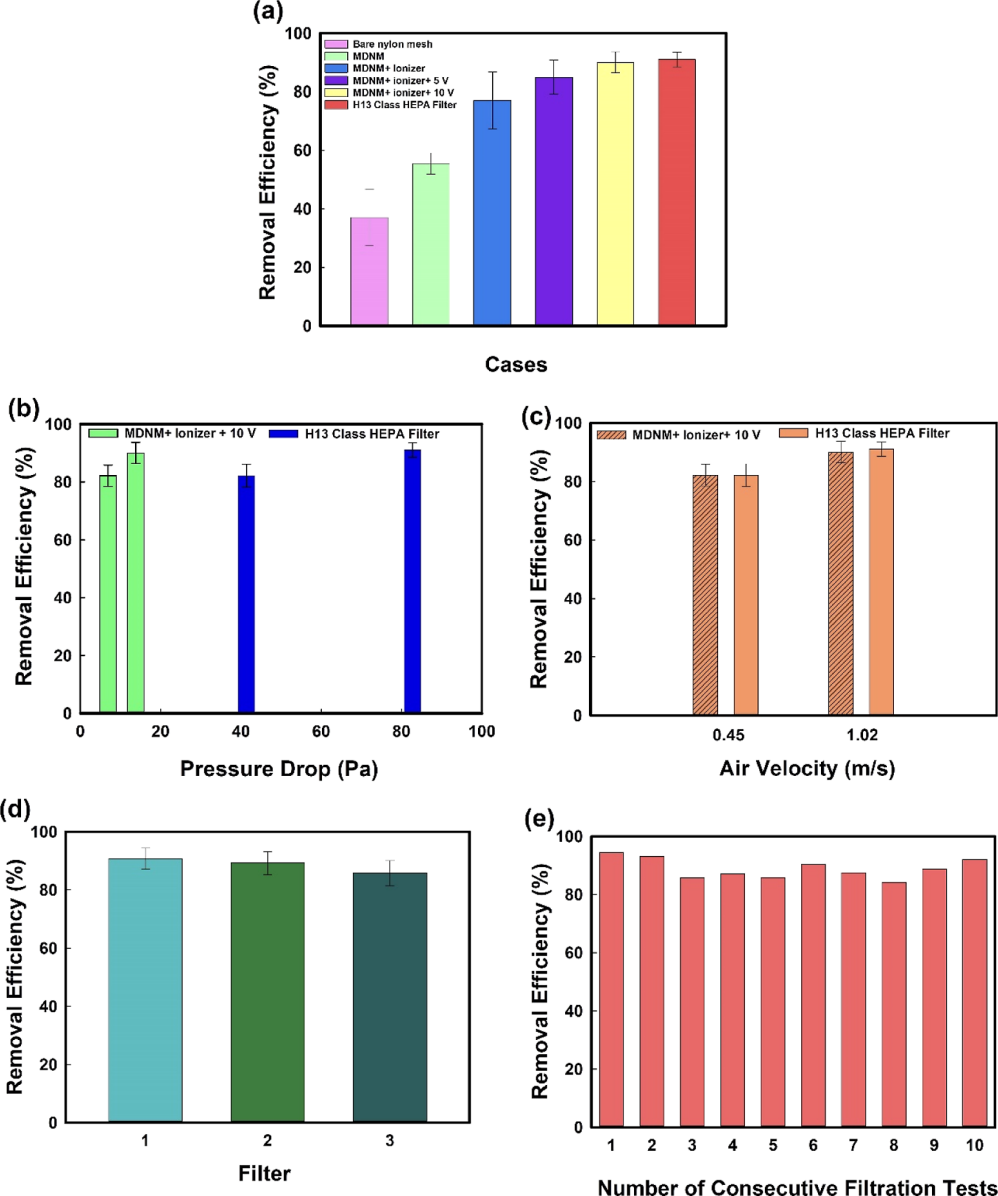
(a) PM_2.5_ removal efficiencies of MDNM filters, (b) PM_2.5_ removal efficiencies with respect to pressure drop, (c) filtration performances of MDNM filters for PM_2.5_ at two different air velocities, (d) repeatability of filtration performance with MDNM filters, and (e) consecutive 2 h filtration test results and performance of MDNM filters after each test.

The removal performance of the MDNM filter under the effect of the ionizer and the applied voltage was verified by simple filtration tests performed using (i) only bare nylon mesh, (ii) (MDNM-decorated) filter, (iii) ionizer with filter, (iv) ionizer with filter (5 V), (v) ionizer with filter (10 V), and (vi) H13 class HEPA filter. Bare nylon mesh had a mass concentration-based removal efficiency of 37.07% for PM_2.5_. As shown in Figure 5a, coating with conductive nanomaterial increased the filtration efficiency of nylon mesh to 55.44% for PM_2.5_. Running the ionizer alone also had an effect on PM removal. However, the best PM_2.5_ removal performance (90.05%) was obtained when the ionizer was on and the applied voltage on the MDNM filter was set to 10 V. This finding was in agreement with other studies in the literature for the highest mass-concentration-based removal efficiency. For instance, Jeong et al. (2017),^33^ Huang et al. (2019),^53^ and Narakaew et al. (2022)^56^ had the highest mass-concentration-based removal efficiencies (99.99, 99.65, and 99.9%, respectively) when they applied 10 V to their Ag NW-decorated nylon mesh filters. However, in contrast, Zhang and Hsieh (2020)^54^ used a voltage as high as 1000 V in their study, where they used polyester as filter material. They also pointed out that the applied voltage for their Ag/TiO_2_-polyester filter is even lower than that for other typical electrostatic filters, such as those used by Li et al. (2018),^65^ Choi et al. (2017),^66^ and Kim et al. (2018),^67^ which were 2000, 10,000, and 20,000 V, respectively. The removal efficiency was enhanced through the interaction between accessible surface terminating groups of positively charged MXene nanosheets and negatively charged PM_2.5_ particles. It should be kept in mind that the applied voltage increases both energy consumption of the filters and the cost of operation. In addition, it might also lead to Joule heating within the filters.

The studies performed with developed filters, not with MXene but with different types of nanomaterials such as Ag NWs, have revealed removal efficiencies in the range of 98–99.99% with a pressure drop in the range of 3.5–14.43 Pa,^33,53−56,68^ which are higher than those obtained in this work. Also, the only study using MXene incorporated PAN fibers showed a removal efficiency of 99.7% with a pressure drop of 42 Pa.^31^ The differences between the literature (Table 1) and this study were attributed to the use of different PM measuring devices, different PM size distribution and flow rate of ambient air, nanomaterial decoration efficiency, nanomaterial characteristics, pore sizes of the filter material, and type of filter material. To minimize these effects, H13 class HEPA filters, which were expected to have a removal efficiency of 99.95% (Figure S2), were tested in the same setup used in this work and operational conditions. As seen in Figure 5a, HEPA filters showed a removal efficiency of 91.03%. This difference in expected and observed removal performance of HEPA filters might be due to the use of a single particle counter while performing the filtration tests as well as the filtration setup used. Nevertheless, filtration tests have confirmed that (regarding the mass-concentration-based removal efficiency calculation values) MDNM filters have very similar efficiencies to those of HEPA filters under the same conditions with the designed filtration setup. Thus, in this respect, MDNM filters might compete with the commercial HEPA filters.

#### 3.2.2 Effect of Air Velocity on the Filtration Performance of MDNM Filters

In addition to the removal efficiency, air permeability is also considered as another main parameter. The term pressure drop is used as a measure of permeability, and it is described as the resistance of filter to airflow.^21,52^ Air permeability is directly related to pressure drop. As the filter is used, it becomes clogged and its permeability decreases, while the resistance of the filter increases and therefore the pressure drop increases. When the filter is clogged, the fan speed should be increased to maintain the same airflow rate and compensate for the increased filter resistance. This causes an increase in the energy consumption.

The filtration performance of air filters varies according to the air velocity and therefore the air flowrate.^69^ A digital pressure manometer was used to measure pressure drop of the filter while an anemometer was used to measure the air velocity in this work. Under low air velocity, low pressure drops were measured as 6.89 and 41.36 Pa for MDNM and HEPA filter, respectively. For high air velocity, pressure drops were measured as 13.78 and 82.73 Pa for MDNM and HEPA filter, respectively. These results are provided as bar charts in Figure 5b. An extremely low-pressure drop was obtained for MDNM filters because the nylon mesh had large openings compared to commercial HEPA filters. Most of the HEPA filters have a pore size of 0.3 μm, which is MPPS. This was the main reason why nylon mesh filters were used in the first place. Second, the thin 2D MXene nanosheets decorated on the surface of the nylon mesh only adhere to the fiber surface and do not clog the pores of the nylon mesh. MDNM filters are very advantageous over commercial HEPA filters used in indoor public places such as hospitals and shopping malls due to their low pressure drops.

The removal efficiencies of MDNM filters were examined at two different air velocities of 0.45 and 1.02 m/s, and the results are provided in Figure 5c. In this study, contrary to the general belief in the literature, it was observed that the removal efficiency increases with the air velocity. This result was obtained both in H13 class HEPA filter, which capture PM with the combination of different filtration mechanisms, and in MDNM filters, which capture PM mainly via electrostatic attraction force along with the different filtration mechanisms. Therefore, considering the air velocities examined here, it can be said that the results obtained in this study are not mainly dependent on the filtration mechanism that changes depending on the air velocity. These results can be attributed to the unsteady air flow pattern, another parameter that may be less effective in determining filtration performance.^70^ The low air velocity may have caused unstable or insufficient airflow in the filtration setup.

#### 3.2.3 Evaluation of Repeatability and 2 h Filtration Performance of MDNM Filters

After determining the optimum voltage value and air velocity for MDNM filters, repeatability of filtration performance with MDNM filters and 2 h filtration performance tests were performed. It is important that air filters with similar filtration performances are easily and reproducibly produced. On the other hand, repeatability refers to having similar properties and filtration performances before and after filtration. Three filters produced under the same conditions were subjected to the same filtration tests and showed very similar filtration performances as in Figure 5d. The resistances of these MDNM filters were also similar and measured as 3.86, 3.93, and 3.84 Ω cm.

The long-term usability of air filters is very important for practical use. One of the reasons is that it is not easy for consumers to replace the commercially available filters that are extensively utilized in public areas. Also, filters that need to be changed frequently cause an increase in the operational cost. The other reason is that long-term exposure to air pollution can lead to a wide range of health problems and environmental effects. In order to predict the long-term performances of the conductive nanomaterial-decorated nylon mesh filters, the developed filters were tested for 2 h under an applied voltage and air velocity of 10 V and 1.02 m/s, respectively. These tests were repeated 10 times. Therefore, the MDNM filter was operated for a total of 20 h. While 20 h is not enough to monitor long-term use of filters, this time can help predict the filter behavior. In Figure 5e, disposable MDNM filters sustainably demonstrate similar removal efficiencies for each filtration test and, in turn, for a total of 20 h. In addition, the absence of pressure drop after each 2 h of filtration test indicated that the permeability of MDNM filters did not decrease, so the pressure drop did not increase. Low pressure drop means low energy consumption and low operating cost. These results may be promising for long-term use of MDNM filters without replacement. However, there is still room for improvement and filtration tests should be performed over longer periods of time.

All these advantages in addition to their simple geometry make MDNM filters promising candidates for indoor air purification. The proposed filters can be used in air purifiers or air conditioners where HEPA filters are frequently used. The applied energy and cost should be taken into account while comparing filters. HEPA filters require powerful pumps to generate sufficient airflow against high pressure drop across the filter. This can be expensive and energy-intensive. The produced MDNM filters showed low pressure drop during filtration tests as discussed. Thus, they did not need powerful pumps to operate. It should also be noted that by using nylons with different mesh sizes, filters can be produced to capture even smaller particles such as PM_1_ (particulate matter with diameters less than 1 μm).

The use of MDNM air filters in different applications was envisioned in this work. MXene-decorated filters can be used in passive air cleaning curtains by people who are sensitive to allergens and want to keep their indoor air as clean as possible. In this way, the fan will not be needed and energy consumption will be reduced further. MDNM filters can also be utilized in window screens. This way dust particles will be kept out of the house as well as flies and insects. In addition, since MXene is known to have antibacterial activity,^53^ MDNM filter would further inactivate the airborne bacteria. The studies on removal efficiency of bacteria in air are limited and remains to be investigated further. MDNM filters can be utilized for antibacterial purposes in the future to expand their applications. The MXene-decorated filters can be also developed for not only laboratory research but also can be developed for industrial and commercial applications. The various settings in which filters can be used to collect particles from the air include, but are not limited to, heating, ventilation and air conditioning systems, manufacturing plants and construction sites, and data centers. For industrial applications, MDNM filters can be used to reduce the pollutant at its source. Application of these filters might be extended for removal of smaller particles such as PM_1_ if nylon mesh filters with smaller pore sizes are decorated with MXene, which remains to be investigated. Since MXene can be easily decorated onto many materials with different openings, the use of nanomaterials can be improved by decorating them onto different materials such as recycled textiles. From an environmental point of view, the developed filters might be beneficial not only for air pollution control but also for waste management. Finally, the voltage application can be extended by choosing materials that can withstand Joule heating caused by high voltage application.

## 4 Conclusions

In this study, MDNM air filters were used to capture airborne particles to improve indoor air quality. Filters decorated with high surface area, conductive MXene nanosheets were easily produced by the dip and drying method. The voltage application improved the performance of the filters. PM was effectively filtered by the electrostatic force between positively charged MDNM and negatively charged particles. It has been seen that the use of MDNM filters and ionizer together is very important for the efficient removal of particles ranging in size from 0.3 to 2.5 μm. A high filtration performance of 90.05% is achieved for PM2.5 at a low pressure drop (∼14 Pa), while the removal efficiency of the HEPA filter used as a control and measured under identical conditions is found as 91.03% with a pressure drop of ∼84 Pa. In addition, the investigated MDNM filters are disposable, thin, make use of cheap nylon, and operate at low air resistances. MDNM filters can be optimized for use in pollutant source control, indoor air pollution control, or outdoor air pollution control. All in all, this is a proof-of-concept study to capture PM_2.5_. More work needs to be done in this area in terms of developing advanced materials and devices to meet the various demands.

## Supporting Information Available

SEM image of Ti_3_AlC_2_ MAX phase, technical datasheet of H13 class HEPA filter, SEM images of MDNM filter after the filtration test, removal efficiency results, and SEM image of bare nylon mesh (PDF)
