# Supplementary material for: MXene-Decorated Nylon Mesh Filters for Improvement of Indoor Air Quality by PM2.5 Filtration
Source: ACS Omega. 2023 Jun 21;8(26):23465–76. doi: 10.1021/acsomega.3c00452 (PMC10323941; doi:10.1021/acsomega.3c00452)
Supplement: Supplementary file 1 — ao3c00452_si_001.pdf [file ao3c00452_si_001.pdf]

Supporting Information

MXene Decorated Nylon Mesh Filters for Improvement of Indoor Air Quality by $PM_{2.5}$ Filtration

Melek Hazal Baskoy[1], Oyku Cetin[2], Serkan Koylan[3], Yaqoob Khan[2], Gurdal Tuncel[1], Tuba Hande Erguder[1], Husnu Emrah Unalan*[,2]

[1] Department of Environmental Engineering, Middle East Technical University (METU), 06800 Ankara, Türkiye

[2] Department of Metallurgical and Materials Engineering, Middle East Technical University (METU), 06800 Ankara, Türkiye

[3] Quantum Solid State Physics (QSP), KU Leuven, Celestijnenlaan 220D, Leuven 3001, Belgium

*Corresponding author: unalan@metu.edu.tr

Contents

Figure S1. The SEM image of Ti_3AlC_2 MAX phase.

Figure S2. Technical datasheet of H13 class HEPA filter.

Figure S3. The SEM images of MDNM filter after filtration test.

Figure S4. (a) Size distribution of the particles in indoor air according to the number concentration. (b) Number-concentration based $PM_{2.5}$ removal efficiencies of MDNM filters. (c) Number concentration-based removal efficiencies of MDNM filters for $PM_{2.5}$ at two different air velocities. (d) Number concentration-based removal efficiencies of replicate MDNM filters, and (e) 2-hour filtration performance of MDNM filters based on number concentration.

Figure S5. (a) Change in the resistance of MDNM filters with respect to the mechanical bending between down to an angle of 80°, (b) Change in the resistance of MDNM filters during10 cycles of Scotch tape test.

Figure S6. SEM image of a bare nylon mesh.

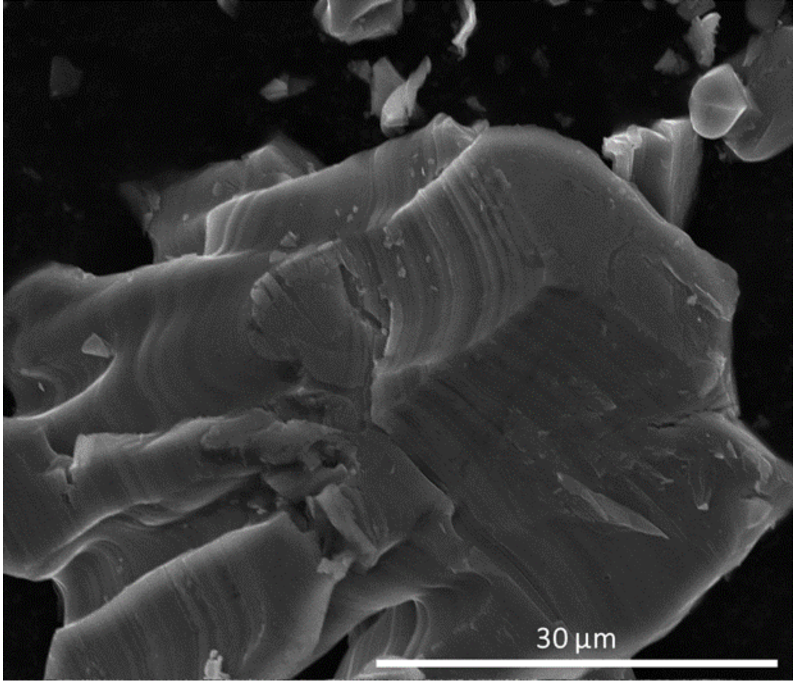

Figure S1. The SEM image of Ti_3AlC_2 MAX phase.

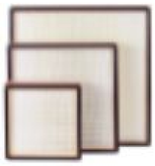

TECHNICAL SPEC. DATASHEET

HEPA FILTER						
Dimensions (mm)			Filter Class (EN 1822)	Filtration Area (m^2)	Nominal Air Flow (m^3/h)	Initial Resistance (Pa)
W	H	D				
140	140	30	H13	0,22	15	100(±10%)

Filtration Media: Micro Glassfiber

Seperator: Thermoplastic Hotmelt

Sealant Type:2K Poly

Frame Type: Galvanized

Final Pressure Drop: 600 Pa (recommended)

Max. Temperature: 70°C

Max. Humidity: %90 rH

Figure S2. Technical datasheet of H13 class HEPA filter used in this work as control.

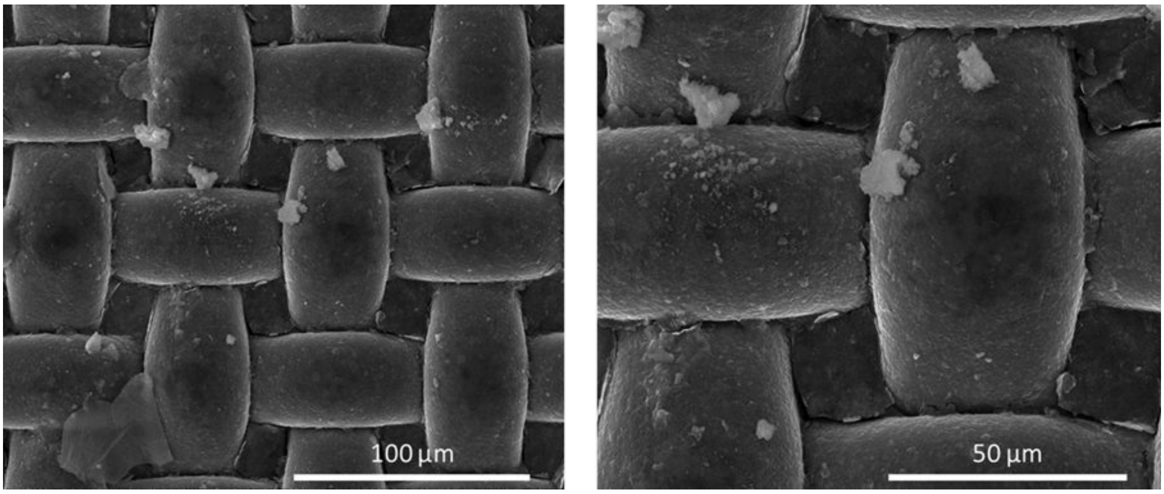

Figure S3. The SEM images of MDNM filter after filtration test.

The relationship between the mass concentration and number concentration can be expressed as (Equation 1):

$$m_{dp_i} = C_f \frac{\pi}{6} {dp_i}^3 n_{dp_i} \qquad (1)$$

,where i denotes channel number of the particle counter; d_{pi} is the arithmetic mean diameter of the upper and lower boundaries for channel i; m_{dpi} is the mass concentration in channel i; n_{dpi} is the number concentration in channel i; and C_f is a correction factor (Cheng and Lin, 2010). Number concentrations were converted into mass concentrations by using an instrument-specific factor (*Cf*) developed by the manufacturer.

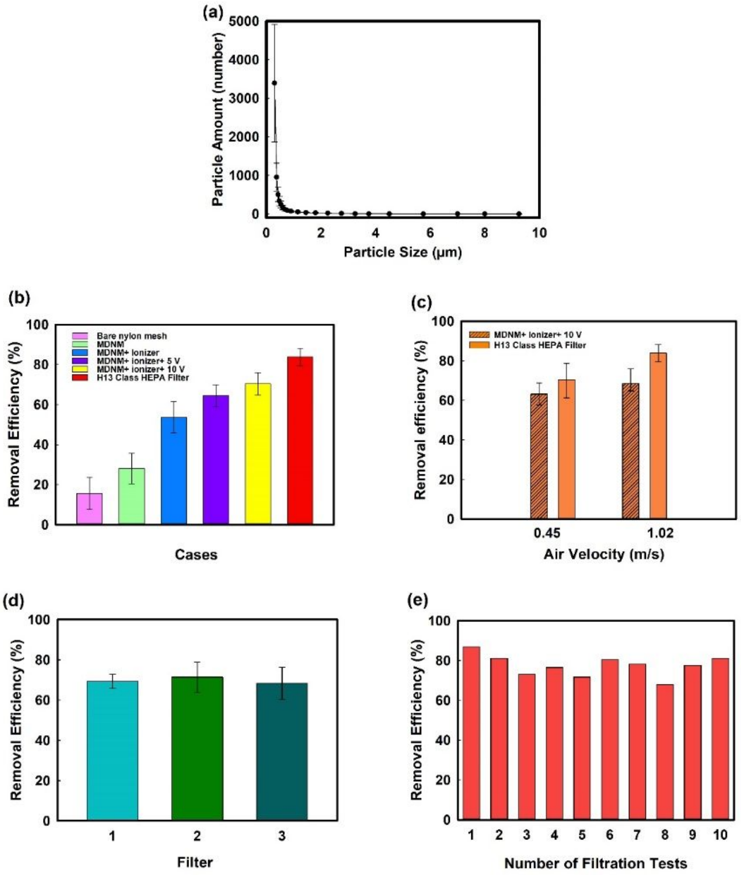

Figure S4. (a) Size distribution of the particles in indoor air according to the number concentration. (b) Number-concentration based $PM_{2.5}$ removal efficiencies of MDNM filters. (c) Number concentration-based removal efficiencies of MDNM filters for $PM_{2.5}$ at two different air velocities. (d) Number concentration-based removal efficiencies of replicate MDNM filters, and (e) 2-hour filtration performance of MDNM filters based on number concentration.

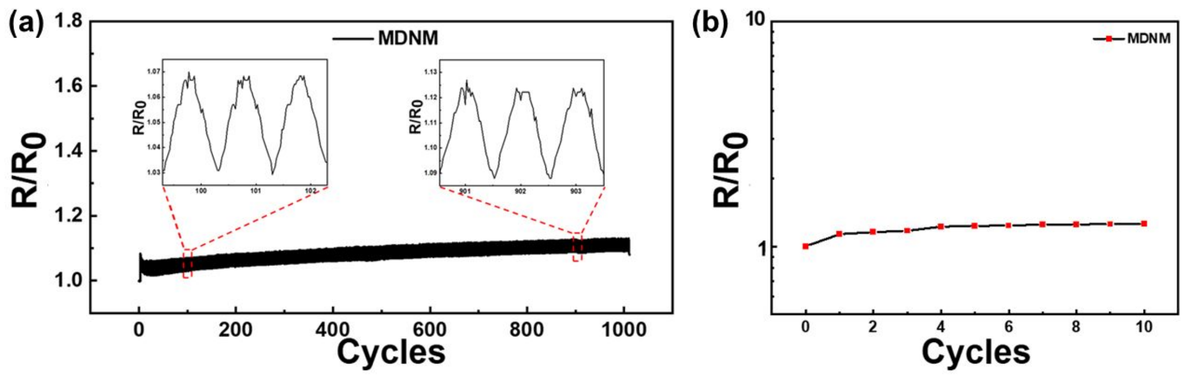

Figure S5. (a) Change in the resistance of MDNM filters with respect to the mechanical bending down to an angle of 80°, (b) Change in the resistance of MDNM filters during10 cycles of Scotch tape test.

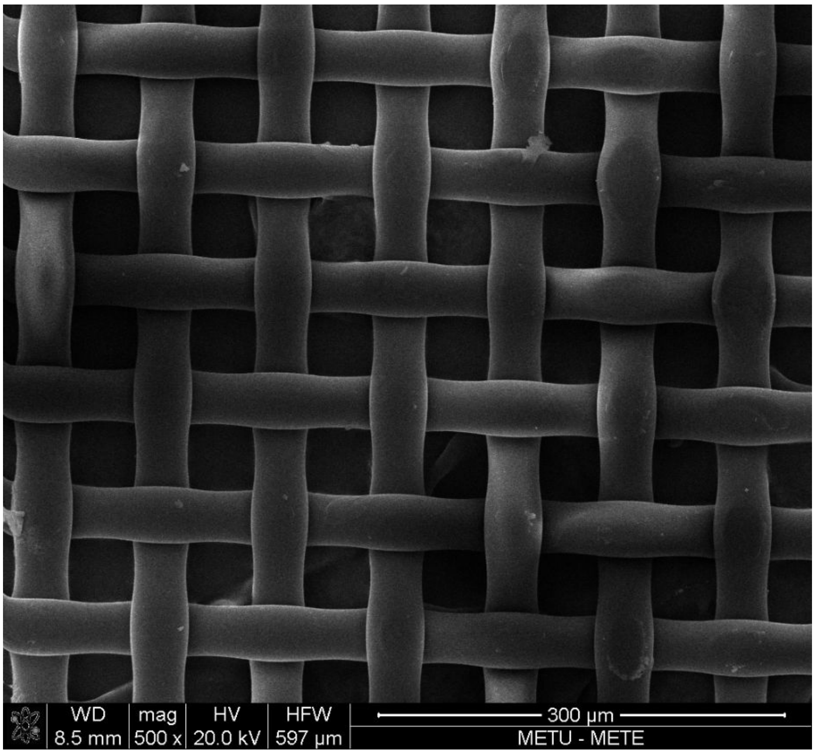

Figure S6. SEM image of a bare nylon mesh.
